# Spatiotemporal trends and drivers of global karst soil erosion based on trend and scenario indicators

**DOI:** 10.1371/journal.pone.0347643

**Published:** 2026-05-05

**Authors:** Yan Yan, Mingxie Chen, Chuanyong Tang, Sheng Wang

**Affiliations:** 1 Key Laboratory of Environment Change and Resources Use in Beibu Gulf, Ministry of Education, Nanning Normal University, Nanning, China; 2 Guangxi Key Laboratory of Earth Surface Processes and Intelligent Simulation, Nanning Normal University, Nanning, China; 3 School of Geography and Planning, Nanning Normal University, Nanning, China; Shandong University, CHINA

## Abstract

Karst ecosystems, 15% of Earth’s land, are critical for soil erosion research due to their unique geology and hydrology. This study investigated the patterns and trends of soil erosion in global karst regions from 2000 to 2020 using the Integrated Valuation of Ecosystem Services and Trade-offs (InVEST) model combined with robust trend methods (Theil-Sen and Mann-Kendall). Correlation analysis and scenario analysis were employed to quantify the driving factors and evaluate the contributions of various factors. A global decline in erosion was found, with intense erosion in southwest Asia, southern Europe, and northwestern North America. Asia had the highest soil erosion rate, followed by North America, Europe, and other regions. Among them, the soil erosion rate in North America showed an upward trend. Russia, China, and Europe were key in erosion reduction (29.98%, 28.01%, 18.68%). Rainfall strongly correlated with erosion; vegetation’s link varied by region. Temperature negatively correlated with erosion in some areas. Scenario analysis quantified contributions of human activities (47.06%) and climate (26.68%). These findings highlight the joint role of natural and human factors in soil erosion management, important for crafting effective conservation strategies in karst areas globally.

## Introduction

Karst regions encompass approximately 15% of the Earth’s continental topography, are inhabited by approximately 17% of the global population [1,2], and are characterized by unique geological formations. Karst landscapes predominantly emerge from soluble carbonate rocks shaped by the erosive forces of the running water. Karst ecosystems are exceptionally vulnerable, with soil typically lying at shallow depths (usually < 10 cm) and scarce surface runoff, compounded by sparse vegetation cover and limited water retention capacity [3], karst ecosystems are exceptionally vulnerable [4]. The confluence of these delicate hydrological attributes and substantial population pressures renders karst regions as focal points for soil erosion. Despite global climate shifts, karst ecosystems confront soil erosion instigated by natural occurrences, including elevated temperatures and intense precipitation, as well as anthropogenic activities, including deforestation, cultivation on steep slopes, and excessive grazing [5]. Soil erosion can lead to alterations in ecosystem patterns, exacerbating the degradation of ecosystem quality and the impairment of ecosystem services. Therefore, a comprehensive appraisal of the status and trajectory of soil erosion in global karst regions is imperative.

Currently, the primary methods used to assess soil erosion levels include the universal soil loss equation (USLE), the Revised Universal Soil Loss Equation (RUSLE), and the integrated valuation of InVEST model. The USLE comprehensively incorporates rainfall, soil characteristics, topography, vegetation cover, and management practices [6]. However, the USLE model lacks consideration for the plot’s ability to intercept upstream sediments. To resolve this limitation, the United States Department of Agriculture and Water Resources created a revised version called the RUSLE [7]. The RUSLE model exhibits a simplified structure, clearer physical meanings of the parameters, and greater accuracy at the watershed level in comparison to the USLE model [8], rendering it a commonly used calculation model for soil erosion. Building on the RUSLE framework, the concept of landscape hydrological connectivity was first introduced by Borselli et al. [9], which was subsequently integrated into the InVEST model. This adaptation enables the simulation of changes in the ecological service system quality and value under various land cover scenarios, effectively capturing soil erosion’s severity and potential impacts. Compared to RUSLE, the InVEST model inherits its strengths and offers enhanced visualization capabilities, comprehensive parameterization, and more intuitive output results through data quantification and spatial visualization [10]. This study selected the InVEST model as the soil erosion calculation model because of its advantages in simulating large-scale ecological service function assessments using the year as the unit. Moreover, further economic cost analysis of soil erosion was required in subsequent research.

Previous studies have revealed the significant spatiotemporal heterogeneity of karst soil erosion, which is caused by intricate internal processes [11]. In recent years, several studies have employed the Universal Soil Loss Equation (USLE) and the Revised Universal Soil Loss Equation (RUSLE) models, in conjunction with the Mann-Kendall test, to perform multi-temporal and spatial analyses of soil erosion in karst regions within the study area. These investigations encompass interannual, seasonal, and interdecadal trends, providing quantitative evaluations of the intensity and magnitude of soil erosion in karst landscapes. [12–15]. However, these research methodologies may have limitations in their applicability when addressing global-scale karst soil erosion issues and often neglect to consider the combined effects of natural and anthropogenic factors comprehensively. Concurrently, certain studies have employed outdated soil-erosion classification standards [16]. Given that karst soils are predominantly composed of insoluble substances [17], the soil formation rate in these regions threatens 28% of global karst ecosystems, covering an area of 594 km^2^. In most karst areas, the content of insoluble materials within the total substrate typically constitutes less than 10%, while pure limestone or dolomite accounts for under 1%. This low proportion of insoluble components results in a slow rate of soil development. Specifically, the formation of a soil layer approximately one meter in thickness on a pure carbonate bedrock is estimated to require 10,000–40,000 years per meter [18]. Consequently, using an excessively high soil erosion rate and outdated universal standards may not be suitable for karst regions, potentially leading to underestimation of the risks of soil degradation and desertification.

The research period from 2000 to 2020 provides a long-term sequential analysis perspective, which helps to reveal the long-term impact of Land Use/Cover Change (LUCC) on soil erosion. This period has witnessed a significant acceleration of global urbanization [19], especially in economically developed areas [20], where the rapid expansion of urban land is often accompanied by a reduction in vegetation cover and an increase in impermeable surfaces. These changes may have intensified the risk of soil erosion. In addition, several countries, including China, have implemented large-scale ecological conservation projects during this time to combat desertification and improve ecological conditions [21]. These policies and ecological management measures may have affected the spatial and temporal distribution of soil erosion [22]. Therefore, studying this time period can provide a more comprehensive understanding of the dynamic changes in soil erosion against the backdrop of global changes, offering a scientific basis for the development of effective soil protection measures.

Analyzing the causes of soil erosion is a crucial step in preventing and controlling it. In previous studies, vegetation cover, topography, and rainfall have been the primary factors influencing soil erosion [23]. The majority of research on the connection between soil temperature and carbon storage has concentrated on how temperature affects soil carbon storage [24,25], neglecting the fact that temperature variations frequently have an indirect impact on soil erosion through changes in rainfall, vegetation growth, economic development, and agricultural practices [26]. Some scholars have suggested that global warming may accelerate the rate of carbonate rock dissolution [27], and that the soil environment is affected by extreme climate events, causing major fluctuations in precipitation [23], which has a direct or indirect negative impact on soil erosion [28]. Because of the limited greening buffer capacity in karst areas [29], more research is needed to understand the connection between soil erosion and plant cover. As an important indicator of regional ecological environmental change, the state of ecosystem services is altered, and soil erosion is directly impacted by LUCC [30,31], which also affects the structure and function of the ecosystem [32,33]. Unreasonable land use methods lead to the deterioration of soil texture, and the aggravation of soil erosion further restricts land use methods and structures, exacerbating land productivity degradation [34]. In karst areas, it remains uncertain what is the relative contribution rate of human factors and climate to soil erosion. Situation analysis offers the benefit of quantifying the relative contribution rate of climate and human factors and illustrating the spatial distribution of each contributing factor, which strongly supports relevant decision-making in soil management. Attribution analysis has been employed in certain studies to quantify the influence of human activities and climate change on soil erosion [35]. Several studies have evaluated the influence of climate change by modeling variations in the distribution of arable land, revealing notable spatial and temporal heterogeneity. Given that regional effects frequently diverge from aggregate global results, certain regions experience more adverse conditions under mitigation scenarios compared to non-mitigation scenarios [36,37].This phenomenon underscores the complex responses of future land use patterns within the framework of climate change and has yet to be applied to research on global karst regions. Hence, it is essential to conduct a thorough investigation into the roles and interrelationships among climate change quantification, land use and land cover alterations, and the global dynamics of karst soil erosion.

This study employed two statistical methods: Theil-Sen Median (a robust nonparametric trend estimator) [38–40] and Mann-Kendall(MK) test (distribution-free significance assessment) for time-series analysis [41,42]. Based on this, the objectives of this study were as follows: (1) To map the spatiotemporal patterns of soil erosion in global karst regions (2000–2020) employing the InVEST model; (2) To elucidate the interannual and seasonal variations in soil erosion and identify trends over the study period; (3) To assess the interplay between soil erosion and its key drivers (rainfall, vegetation cover, and land surface temperature), and to quantify the relative impacts of climate change and human activities on erosion dynamics.This study helps to better understand the key influencing factors of soil erosion in karst regions, provides empirical support for global sustainable soil erosion management and the layout of karst ecological restoration, and promotes green and sustainable development.

## Materials and methods

### Study area

**Fig 1:**
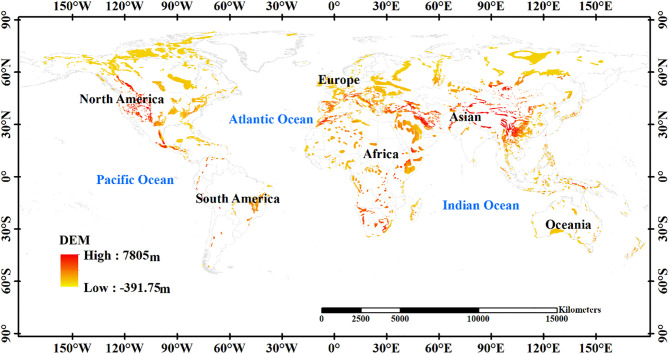
Global distribution of karst areas.

This investigation examined the global distribution of karst regions, with an area of approximately 2.2 × 10^7^ km^2^ (Fig 1). The Karst Science Data Center was the source of the global karst region data (http://geoinfo.irck.org.cn:8099/portal/index). Karst landforms are present in a wide range of regions worldwide, such as the Dinaric Alps, the Central Plateau of France, the Ural Mountains in Russia, the southern part of the Australian continent, Kentucky and Indiana in the United States, Cuba, Jamaica, and northern Vietnam [43].

### Study framework

**Fig 2:**
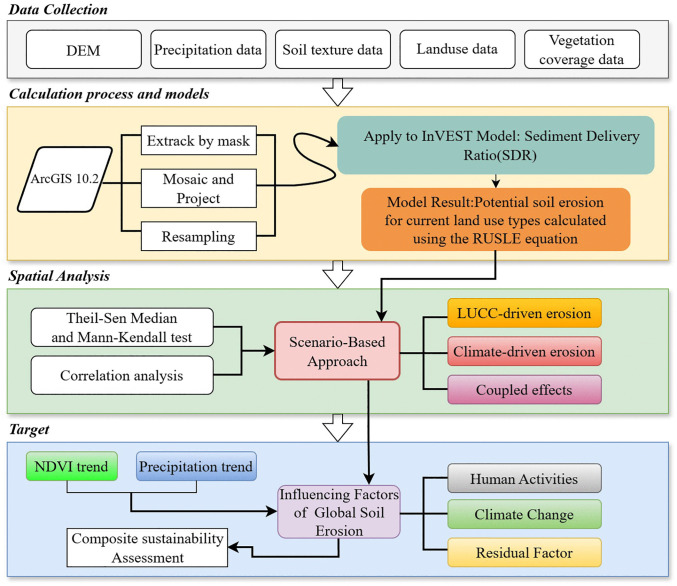
Method framework of the study.

To ensure a clear and coherent research workflow, the overall process of this study is illustrated in the workflow diagram (Fig 2). The research begins with the collection and preprocessing of raw datasets, including DEM, precipitation data, land use data, soil texture data, and vegetation coverage data. Subsequently, the raw data are preliminarily processed using ArcGIS 10.2. The processed datasets are then imported into the InVEST model, and the results derived from the modified RUSLE equation are integrated with Theil–Sen trend analysis, the Mann–Kendall test, and spatial autocorrelation methods to identify global karst soil erosion patterns. Following this, a scenario analysis approach is applied, in which the above analytical results are overlaid to accurately assess the contribution rates of various factors to global soil erosion patterns. This provides scientific support for land-use optimization and informed decision-making.

### Datasets sources

This research employed the sediment delivery ratio (SDR) component of the InVEST model, version 3.14.0. Comprehensive data pertaining to precipitation, soil properties, land use, digital elevation models, and vegetation cover are provided in Table 1. Data preprocessing began with importing the data into ArcGIS 10.8, and the coordinate system was defined as WGS1984 via projection. Subsequently, all datasets were resampled to achieve a unified spatial resolution of 1 km × 1 km, laying the foundation for the basic map of the study area in subsequent analyses.

**Table 1:** Source and explanation of basic data.

Data Type	Data Source	Meaning of Data usage
Digital Elevation Model (DEM)	CGIAR Consortium for Spatial Information(https://srtm.csi.cgiar.org)	Deriving terrain factors (LS)
Precipitation data	European Centre for Medium-Range Weather Forecasts (https://cds.climate.copernicus.eu/cdsapp)	Calculating rainfall erosion factor (R)
Soil texture data	Food and Agriculture Organization of the United Nations (https://www.fao.org/soils-portal/)	Calculating the K factor for indicating soil erodibility
Land use data	European Space Agency (https://maps.elie.ucl.ac.be/CCI/viewer/download.php)	Calculating the P factor
Vegetation coverage data	(MODIS MOD13Q1, 250m) from NASA (https://lpdaac.usgs.gov/).	Calculating the C factor
Land Surface Temperature data	(MOD11A1 V6, 1Km) from NASA (https://lpdaac.usgs.gov/).	Analyze the correlation withsoil erosion

### Methods

#### RUSLE model.

The InVEST Sediment Delivery Ratio (SDR) module was employed, integrated with a modified the soil erosion potential (A) is calculated using the Revised Universal Soil Loss Equation (RUSLE) framework. The RUSLE formula is expressed as:


A=R×K×L×S×C×P (1)

Key parameters include: R-factor: Derived from ERA5 precipitation data [44,45], representing the rainfall erosivity index; K-factor: Calculated using the Wischmeier equation [46], indicating soil erodibility; C-factor: Regression-based vegetation coverage coefficient [47], reflecting the effect of vegetation on soil protection; P-factor: Assigned by land use classification [6], representing the impact of soil conservation practices.

Owing to the distinct climatic and geomorphic characteristics of karst regions, such as exceptionally slow soil formation rates and high landscape sensitivity to erosional processes that trigger sinkhole formation, conventional soil erosion thresholds are inadequate [48]. To address terrain instability and irreversible bedrock exposure risks specific to karst systems, we adopted a modified six-tier classification system based on annual erosion modulus: Minimal (<500 t·km^-^²·a^-^¹), Mild (500–2500 t·km^-^²·a^-^¹), Medium (2500–5000 t·km^-^²·a^-^¹), Intense (5000–8000 t·km^-^²·a^-^¹), Extreme (8,000–15,000 t·km^-^²·a^-^¹), and Severe (>15,000 t·km^-^²·a^-^¹). This framework, referenced from prior studies [49], enforces stricter limits aligned with the ecological fragility of karst landscapes.

#### Theil-Sen Median and Mann-kendall test.

Theil-Sen trend estimation constitutes a non-parametric approach employed to determine the trend line within time series data. This method calculates the slope of the trend line by taking the median of the slopes derived from all possible pairs of data points.


$$\beta =median\left(\frac{x_{j}-x_{i}}{j-i}\right),\;\forall j>i$$ (2)

In the given formula (2), the variables *x*_*j*_ and *x*_*i*_ correspond to the *j*-th and *i*-th observations of the time series *x*_*t*_ respectively. The *β* represents the slope coefficient, where a positive value *β* > 0 signifies an increasing trend, and a negative value *β* < 0 indicates a decreasing trend in the time series throughout the study period.

The Mann-Kendall test is a non-parametric statistical procedure designed to identify the existence of a trend in time series datasets [50,51] and it was defined as follows:


$$S=\sum_{i=\text{1}}^{n-\text{1}}\sum_{j=i+\text{1}}^{n}sgn\left(x_{j}-x_{i}\right)$$ (3)

$$sgn\left(x_{j}-x_{i}\right)=\left\{ \begin{array}{c} \text{1},\\ \text{0},\\ -\text{1}, \end{array}\;\begin{array}{c} {\;x}_{j}-x_{i}>\text{0}\\ \;x_{j}-x_{i}=\text{0}\\ \;x_{j}-x_{i}<\text{0} \end{array}\right.\;$$ (4)

$$Z=\left\{ \begin{array}{cc} \frac{S-\text{1}}{\sqrt{\text{Var}(S)}}, & S>\text{0}\\ \text{0}, & S=\text{0}\\ \frac{S+\text{1}}{\sqrt{\text{Var}(S)}}, & S<\text{0} \end{array}\right.\;$$ (5)

*S* represents the full collection of statistics obtained from the analyzed time series, whereas Var(*S*) denotes the variance corresponding to *S*. Trends were deemed statistically significant when the absolute value of Z exceeded 1.65, 1.96, or 2.58, corresponding to confidence levels of 90%, 95%, and 99%, respectively (*α* = 0.05).

#### Correlation analysis.

To investigate the association between global karst soil erosion and meteorological variables, specifically temperature and precipitation, in addition to vegetation cover, the Pearson correlation coefficient (R) was computed using the following formula:


$$=\frac{\sum_{i=\text{1}}^{n}\left(x_{i}-\overset{―}{X}\right)\left(y_{i}-\overset{―}{Y}\right)}{\sqrt{\sum_{i=\text{1}}^{n}{\left(x_{i}-\overset{―}{X}\right)}^{\text{2}}}\sqrt{\sum_{i=\text{1}}^{n}{\left(x_{i}-\overset{―}{Y}\right)}^{\text{2}}}}$$ (6)

The variable *r* denotes the correlation coefficient between variables *x* and *y*, with its values spanning from −1–1. The terms *x*_*i*_ and *y*_*i*_ represent the observed values for the $i^{t\text{h}}$ year, while $\overset{―}{X}$ and $\overset{―}{\overset{―}{Y}}$ correspond to their respective mean values calculated over a 21-year period.

Pearson’s correlation to quantify variable relationships (rthresholds: 0.8–1.0 = extremely strong, 0.5–0.8 = strong, 0.3–0.5 = moderate, < 0.3 = negligible), calculated using standard mean-centered formulae.

#### Determination of the contribution rates of various influencing factors to soil erosion.

To quantify the relative contributions of climate change (ΔCli), land use/cover change (ΔLUCC), and residual factors (ΔR) to soil erosion dynamics, we employed a scenario-based approach [52] combined with decomposition formulae (Eqs.7–15).It should be emphasized that scenario analysis is a quantitative partitioning of observed changes under the model structure and scenario assumptions (e.g., simulation settings where one factor is fixed while others vary over time). It reflects the relative contribution/explanatory power of each factor to the simulated changes under the selected model and scenarios. Conclusions from scenario analysis depend on input data, model parameterization, and the ceteris paribus assumption; therefore, the results should be interpreted as attributional/explanatory evidence under given assumptions, rather than definitive causal relationships without additional constraints.

Three scenarios were designed: (1) LUCC-driven erosion (fixed 2000 climate with 2000–2020 LUCC data), (2) climate-driven erosion (fixed 2000 land use with 2000–2020 meteorological data), and (3) coupled effects (both variables evolving synchronously). The total erosion change (ΔErosion) was partitioned into ΔCli, ΔLUCC, and ΔR, with their relative contributions (Con_C_, Con_L_, Con_R_) calculated as ratios to the sum of absolute values, enabling isolation of individual and interactive impacts.


$$\Delta \text{Erosion}={\text{Erosion}}_{\text{j}}\times S_{\text{j}}-{\text{Erosion}}_{\text{i}}\times S_{\text{i}}$$ (7)

$$\Delta {\text{Erosion}}_{\text{c}}={\text{Erosion}}_{\text{i}}\times (S_{\text{j}}-S_{\text{i}})$$ (8)

$$\Delta {\text{Erosion}}_{\text{L}}=({\text{Erosion}}_{\text{j}}-{\text{Erosion}}_{\text{i}})\times S_{\text{i}}$$ (9)

$$\Delta \text{Cli}=\Delta \text{Erosion}-\Delta {\text{Erosion}}_{c}$$ (10)

$$\Delta \text{Lucc}=\Delta {\text{Erosion}-\Delta \text{Erosion}}_{\text{L}}$$ (11)

$$\Delta \text{R}=\Delta \text{Erosion}-\Delta {\text{Erosion}}_{c}-\Delta {\text{Erosion}}_{L}$$ (12)

$${\text{Con}}_{\text{c}}=\frac{△\text{Cli}}{\left|△\text{Cli}|+|△\text{LUCC}|+|△\text{R}\right|}$$ (13)

$${\text{Con}}_{\text{L}}=\frac{△\text{Lucc}}{\left|△\text{Cli}|+|△\text{LUCC}|+|△\text{R}\right|}$$ (14)

$${\text{Con}}_{\text{R}}=\frac{△\text{R}}{\left|△\text{Cli}|+|△\text{LUCC}|+|△\text{R}\right|}$$ (15)

## Results

### Spatial distribution patterns of soil erosion intensity levels

**Fig 3:**
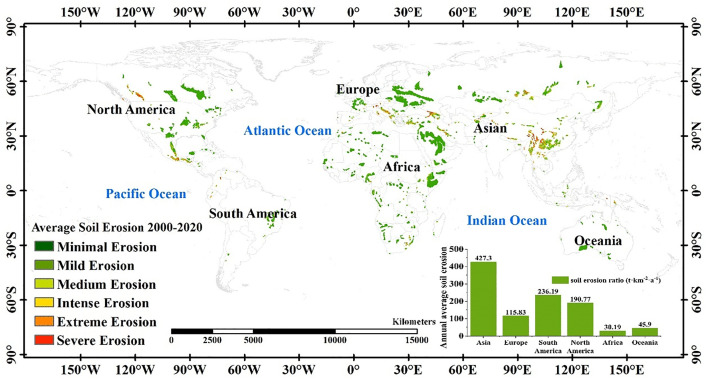
Distribution of average soil erosion levels in karst regions of the world during 2000-2020.

Based on the total soil erosion output of raster cells generated by the InVEST-SDR model, the spatial distribution pattern of soil erosion intensity in global karst areas was classified. Between 2000 and 2020, the average annual soil erosion in karst regions worldwide totaled 12.38 × 10^8^ t/a. The predominant erosion type observed in these regions was mild erosion, covering 78. 13% of the total karst area globally (Fig 3). Moderate and severe erosions comprised 6.71% and 5.14% of the total area, respectively. Upon examining the distribution of severe soil erosion, it became evident that these areas are concentrated along the edge of the Qinghai-Tibet Plateau in Asia, the high mountain regions of eastern North America, and the mountainous regions bordering Asia, Africa, and Europe.These locations significantly overlapped with high-elevation areas, as depicted in the DEM.

Regarding the spatial distribution, the average soil erosion rate varied across continents with karst terrain. Africa exhibited the lowest erosion modulus at 30.19 t/(km^2^·a), whereas Asia recorded the highest at 427.30 t/(km^2^·a). The sequence of the erosion modulus from highest to lowest was Asia, South America, North America, Europe, Australia, and Africa. The ranking of the proportion of mild erosion was slightly different. The area of mild erosion in Asia accounted for 64.97% of the total area of each level, followed by 75.54%, 78.69%, 82.97%, 89.57%, and 95.67% in South America, North America, Europe, Africa, and Australia, respectively. From the average annual total soil erosion perspective, Asia was the highest, with 8.21 × 10^8^ t/a, and the lowest value in Europe, only 1.25 × 10^8^ t/a.

**Fig 4:**
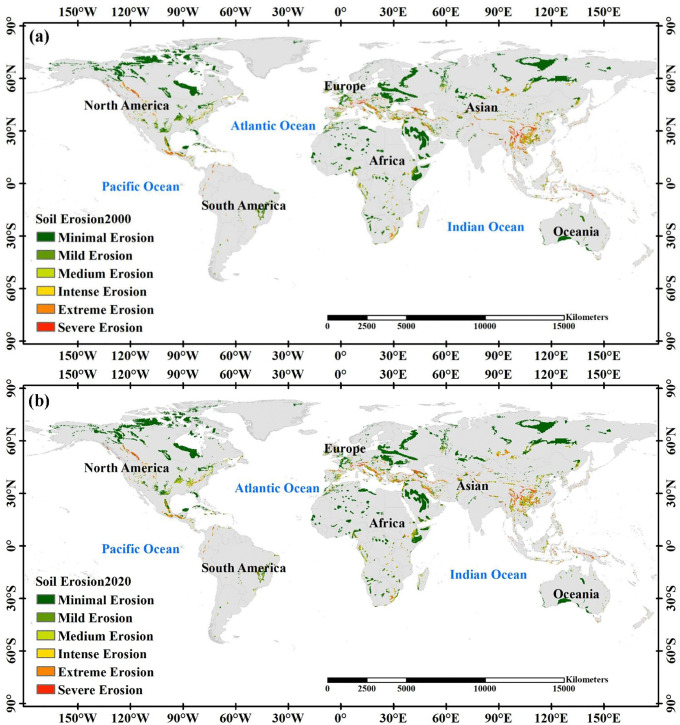
Spatial differentiation of world karst soil erosion levels in 2000(a) and 2020(b).

Comparing the proportion of different levels of soil erosion in the world’s karst areas between 2000 and 2020 revealed a clear developmental trend from mild to more severe erosion and, conversely, from severe to milder erosion. In 2000 (Fig 4a), mild erosion accounted for 78.70% of the total erosion; In 2020(Fig 4b), this proportion decreased to 71.56%. This decline suggests that areas previously considered safe from erosion have suffered degradation. Conversely, severe erosion has decreased from 1.97% in 2000 to 1.45% in 2020, indicating successful erosion control measures in some regions.

### Temporal variations of soil erosion in the global karst region

**Fig 5:**
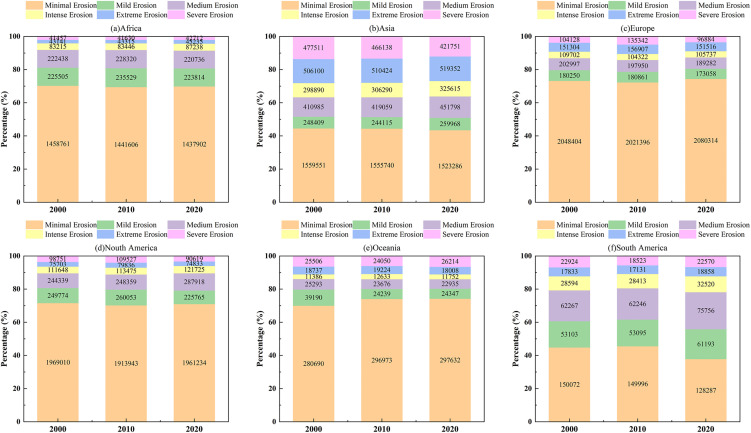
Distribution of soil erosion levels from a global perspective.

Trend and correlation analyses of global karst soil erosion were conducted based on the total potential soil loss per grid cell generated by the InVEST-SDR model. Between 2000 and 2020, soil erosion across six continents demonstrated significant regional disparities. In Africa (Fig 5a), the total area affected by erosion decreased marginally by 0.99%, from 2.01 million km² to 1.99 million km². This reduction was primarily driven by a decline in areas experiencing minimal erosion, which accounted for 1.44 million km² in 2020, while regions subjected to severe erosion remained relatively constant at 41,000 km², indicating a generally low risk of erosion on the continent. Asia (Fig 5b) experienced a 2.3% reduction in minimal erosion area, from 1.56 million km² to 1.52 million km², alongside a substantial decrease in severe erosion by 11.68%, from 477,500 km² to 421,700 km², representing the most pronounced improvement among the six continents. In Europe (Fig 5c), erosion levels remained relatively stable, with minimal erosion dominating at 2.08 million km² in 2020, and The area subject to severe erosion decreased to 96,900 km², reflecting consistent erosion conditions. Erosion patterns in North America show slight fluctuations (Fig 5d). Notably, the area affected by severe erosion decreased from 98,751 km² in 2000–90,619 km² in 2020, representing a decline of 8.23%. However, some moderate erosion classes exhibit a significant upward trend, which may lead to an increase in severe erosion in the future and thus requires close attention. Oceania was predominantly affected by minimal erosion, reaching 297,600 km² in 2020. However, severe erosion increased by 2.3%, from 25,500 km² to 26,100 km², indicating a deteriorating trend relative to other continents (Fig 5e). South America (Fig 5f) experienced fluctuations. The minimum erosion area reached 128,287 km² in 2020, representing a decrease of 14.51% compared with that in 2000. However, erosion in some intermediate classes has also intensified.

**Fig 6:**
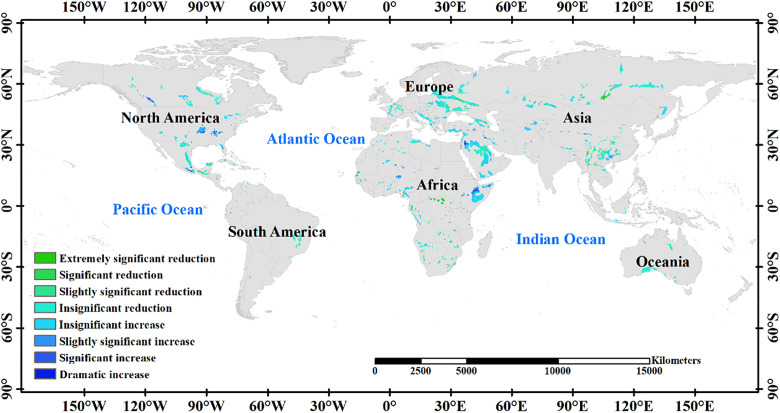
Temporal spatial changes of soil erosion in karst regions of the world from 2000 to 2020.

Over the two decades from 2000 to 2020, areas experiencing an overall decrease in soil erosion covered 52.07% of the total area, slightly surpassing those with an overall increase of 47.93% (Fig 6). Among these, regions exhibiting significant decreases and increases represented 2.70% (*P* < 0.01) and 4.20% (*P* < 0.05), respectively. Conversely, the regions witnessing significant increases and decreases were 4.35% (*P* < 0.05) and 1.00% (*P* < 0.01), respectively. Significant increases in soil erosion from 2000 to 2020 were mainly concentrated in Saudi Arabia, Ethiopia, Canada, the United States, and Mexico, whereas significant decreases were predominantly observed in Russia, China, Europe, and Congo.

**Fig 7:**
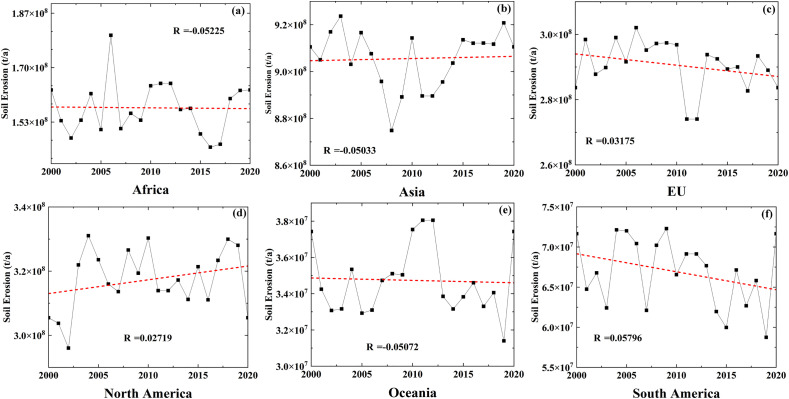
Interannual variation of total soil erosion across six continents from 2000 to 2020(a:Africa; b:Asia; c:EU; d:North America; e:Oceania; f:South America).

Between 2000 and 2020, soil erosion patterns across the six continents demonstrated markedly diverse trajectories, as illustrated in Fig 7. North America exhibited a discernible increase in soil erosion over this period, whereas Asia, Africa, and Oceania did not present any statistically significant upward or downward trends. Conversely, both South America and the European Union (EU) experienced reductions in soil erosion. In Africa, soil erosion levels fluctuated substantially, reaching a peak in 2005 and a minimum in 2016 (Fig 7a), with a correlation coefficient (R = −0.05225) indicating a weak temporal association. Similarly, Asia showed pronounced variability in soil erosion without a definitive linear trend, as evidenced by a low correlation coefficient (Fig 7b). Europe displayed a modest declining trend (Fig 7c), with a weak negative correlation (R = −0.03175). In North America(Fig 7d), soil erosion gradually increased over time, although the linear correlation remained weak (R = 0.0279). Oceania (Fig 7e) experienced significant fluctuations without a clear directional trend. In contrast, South America (Fig 7f) demonstrated a notable downward trend in soil erosion. Collectively, these findings indicate substantial intercontinental variability in soil erosion trends from 2000 to 2020, with most regions exhibiting weak linear correlations with temporal progression.

Based on the significance tests (P < 0.05) evaluating changes over the 21 years, among the top 10 countries with karst areas, Russia, China, and Europe emerged as the top three contributors to soil erosion reduction, both in terms of area and overall impact on the global karst region (Table 2). Considering the diverse countries comprising Europe and the varied sizes of European regions, except for Russia, other European countries were collectively analyzed to facilitate a comprehensive assessment of karst-related phenomena. It is noteworthy that among the ten countries, China, with 7.00% of the global karst area, exhibited the most significant performance in the proportion of soil erosion reduction, reaching 19.33%, becoming the main contributor to the significant reduction of soil erosion in the global karst region.

**Table 2:** Top 10 karst soil erosion Country.

Country	Karst Area (km^2^)	Areas showing decrease	Areas showing increase	Contribution rate	Contribution ranking
Russia	1931825. 67	19. 22%	0. 52%	29. 98%	1
Canada	1601205. 13	3. 33%	8. 22%	0. 52%	6
China	1121944. 83	19. 33%	4. 25%	28. 01%	2
United States	905863. 79	0. 80%	18. 18%	−9. 21%	10
EU	835650. 37	12. 33%	1. 28%	18. 68%	3
Saudi Arabia	612979. 41	2. 33%	6. 77%	−0. 23%	7
Mexico	606455. 46	2. 83%	8. 58%	−0. 48%	8
Australia	343070. 6	0. 42%	0. 14%	0. 57%	5
Brazil	271195. 98	3. 00%	0. 14%	4. 65%	4
Iran	255116. 45	0. 19%	4. 30%	−2. 17%	9

Note: Due to the vast total karst area and the relatively small size of several EU member states, the countries in the EU are considered collectively. *P* = 0.05 was used to indicate statistical significance for the proportion of areas showing a decrease or increase.

**Fig 8:**
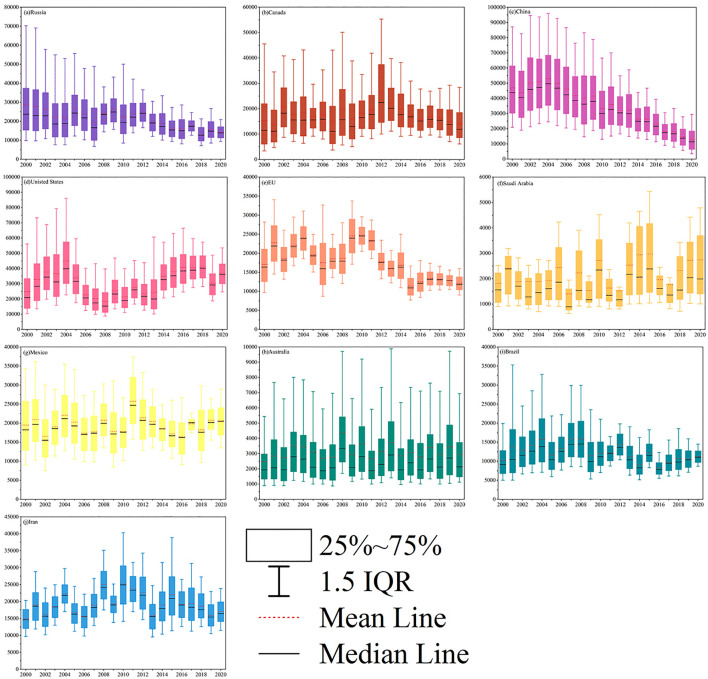
Distribution of soil erosion rates in the top ten countries/region with the largest karst areas around the world during the period 2000–2020.

The horizontal distribution of soil erosion values for individual soil pixels was examined using box plots, based on actual soil erosion output data from the top ten countries or regions with the largest karst areas. Overall, countries demonstrating substantial net reductions in soil erosion are predominantly situated in Asia (e.g., China), Europe (e.g., Russia and the European Union), and South America (e.g., Brazil), which corresponds with the ranking of the top ten global karst countries or regions by contribution rate as presented in Table 2. Prior to 2010, Russia (Fig 8a) and China (Fig 8c) displayed a broad range and high variability in soil erosion data, characterized by large disparities between maximum and minimum values and taller box plots indicative of greater dispersion. Following 2010, both the box heights and interquartile ranges (IQR) of soil erosion values in these countries diminished, suggesting a more concentrated distribution of data and enhanced regulation of erosion levels. In contrast, it is noteworthy that, despite the United States possessing a considerable karst area (Fig 8d), its soil erosion values remained relatively stable at elevated levels throughout the study period, exhibiting no significant temporal change.

### Driver analysis

**Fig 9:**
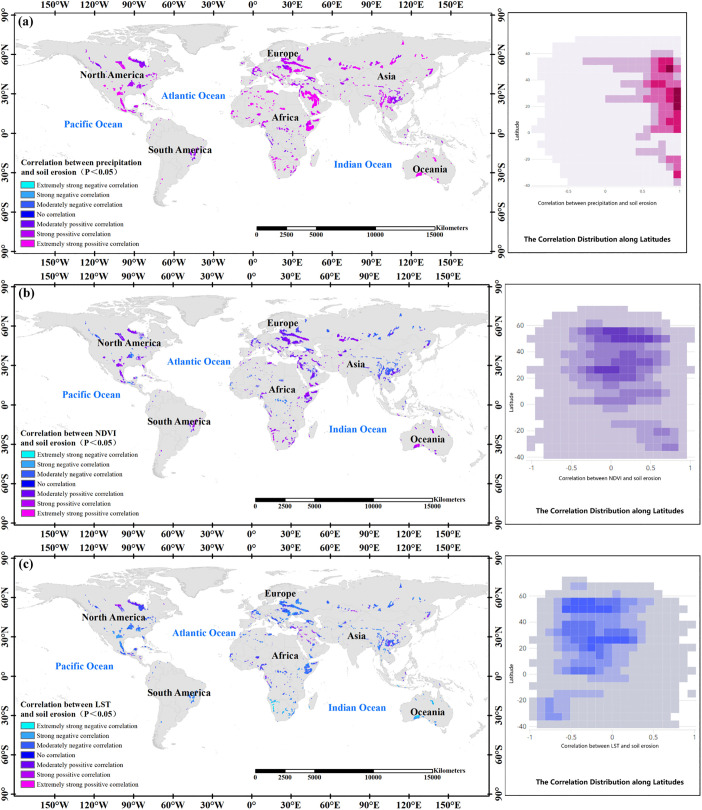
Environmental correlations of precipitation(a), NDVI(b), and land surface temperature(c) with soil erosion.

Soil erosion and rainfall in karst regions are strongly linked, as evidenced by a robust positive correlation (Fig 9a). Areas with an extremely significant positive correlation (*P* < 0.01) covered 81.57% of the total area, whereas those with a significant positive correlation (*P* < 0.05) encompassed 4.53% of the total area. The correlation between rainfall and soil erosion followed a latitudinal pattern, with a significant enhancement near the 30-degree north and south latitudes. However, exceptions include, such as southwest China, where the correlation between rainfall and soil erosion is slightly weaker despite being within the same latitude range.

The relationship between vegetation coverage and soil erosion appeared weak (Fig 9b). In the world’s karst regions, 58.59% of the area does not exhibit a significant correlation between soil erosion and the Normalized Difference Vegetation Index (NDVI) in correlation analysis. Among the remaining 41.41% of the area with a correlation, the impact of NDVI on soil erosion displays spatial variability. Overall, 10.94% of the world’s karst regions displayed a significant positive correlation (*P* < 0.01), whereas 9.13% demonstrated a significant positive correlation (*P* < 0.05). A small portion of the area exhibited a negative correlation, including 1.11% with a significant negative correlation (*P* < 0.01), and 2.42% with a significant negative correlation (*P* < 0.05).

The relationship between surface temperature and soil erosion was generally found to be weak (Fig 9c), with 52.89% of global karst areas showing no significant correlation between these variables. In regions with correlation, Land Surface Temperature (LST), and soil erosion mainly exhibited a negative correlation, meaning higher surface temperatures corresponded to less soil erosion. Specifically, 1.44% of the world’s karst regions exhibited a significant negative correlation (*P* < 0.01), while 12.82% displayed a significant negative correlation (*P* < 0.05), and 17.38% demonstrated a non-significant negative correlation.

In summary, the findings of this study indicate that soil erosion in global karst areas is predominantly affected by temperature and precipitation. Conversely, the anticipated correlation between vegetation cover and soil erosion was not substantiated by the results.

**Fig 10:**
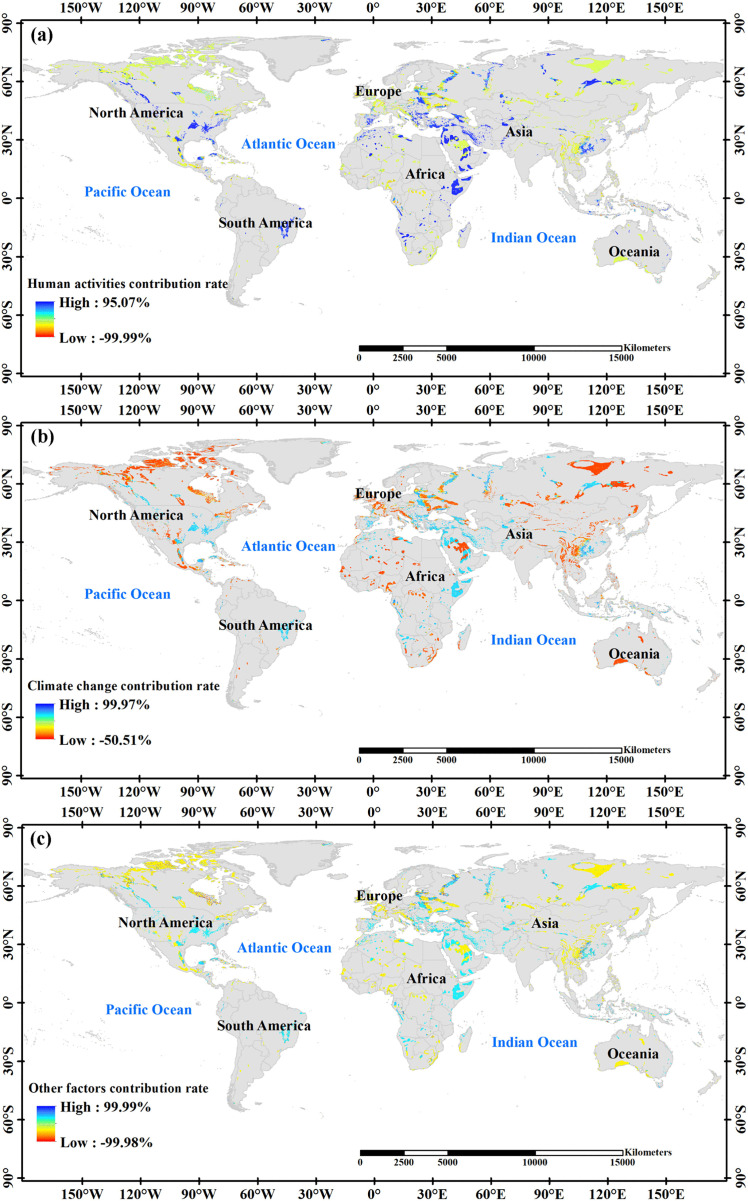
Contribution rates to soil erosion by human activities(a), Climate change(b) and Other factors(c).

The results of the scenario analysis underscore the significant influence of land use changes driven by human activities on soil erosion dynamics (Fig 10a), accounting for 47.06% of the observed variations. Across different continents, human activities predominantly mitigated soil erosion in Australia, Europe, and North America, whereas they generally contributed to an increase in soil erosion in South America.Climate change emerges is another significant factor influencing soil erosion, contributing 26. 68% to the overall variation (Fig 10b). Climate change negatively contributes to southern Australia, indicating its role in inhibiting soil erosion. Conversely, in Europe, climate change predominantly contributed positively. These impacts of climate change vary regionally, with mainly negative contributions observed in Asia, Australia, Europe, and North America and predominantly positive contributions in Africa and South America.The dynamics of soil erosion (Fig 10c) are also affected by other residual factors, including land use changes, which contribute 26.26% to the observed changes. Compared to other continents, Europe, northern North America, and southwestern China experienced a significant decrease in soil erosion, while eastern North America experienced a significant increase in soil erosion.

Although precipitation shows a significantly positive correlation with erosion in 81.57% of the study area, indicating that precipitation effects are spatially widespread, scenario decomposition reveals that land use/cover change contributes more to the overall erosion change. This is because land use change tends to be concentrated in erosion-sensitive areas or areas with high baseline erosion rates, exerting an amplifying effect on the total amount. Meanwhile, the impact of precipitation on erosion in many pixels appears as short-term or seasonal fluctuations, with high correlation but limited contribution to long-term trends, whereas land use change usually exhibits a persistent trend and is thus more significant in terms of cumulative trend contribution. Furthermore, precipitation can also indirectly affect erosion through vegetation (C factor), and this indirect effect is assigned to the land use/cover component in the contribution allocation during scenario decomposition.

**Fig 11:**
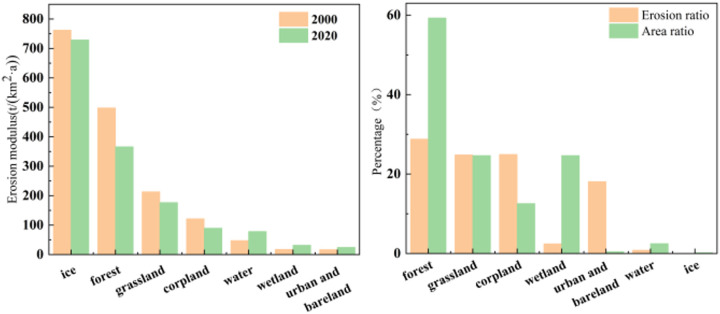
Soil erosion characteristics across various land use types.

**Fig 12:**
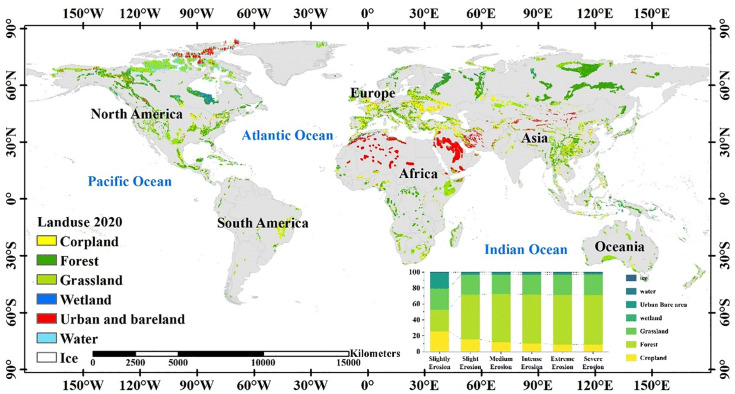
Distribution of land use types and soil erosion levels in karst areas of the world in 2020.

An analysis of soil erosion severity in relation to land use categories indicates that regions experiencing intense erosion are predominantly linked to cultivated lands, urban construction areas, and bare soils (Fig 11). The magnitude of soil erosion demonstrates considerable variation across different land use types (Fig 12), each characterized by unique patterns of erosion severity. Among these, forests and wetlands exhibit the lowest levels of soil erosion. The root networks of forest trees, combined with the protective canopy layer, play a crucial role in reducing soil erosion caused by water runoff, although additional influencing factors warrant comprehensive examination. Conversely, grasslands show relatively uniform distributions across erosion severity levels, suggesting a reduced susceptibility to variations in erosion intensity.

## Discussion

### Variation of soil erosion

**Fig 13:**
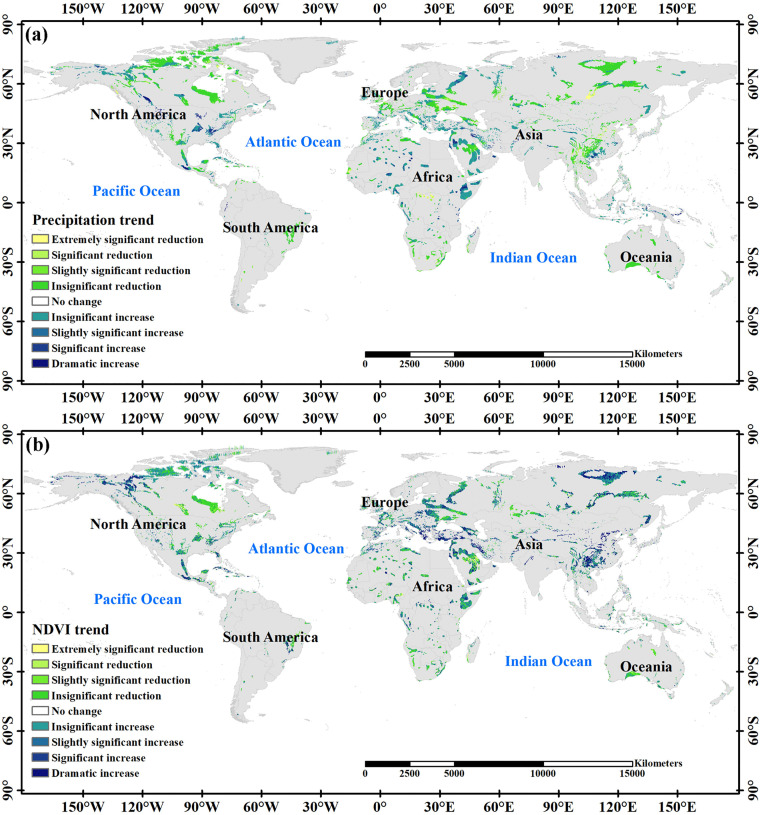
Precipitation(a) and NDVI(b) trends in karst regions of the world between 2000 and 2020.

A significant correlation existed between soil erosion and changes in rainfall patterns. Regions experiencing increased rainfall coincide with areas of intensified soil erosion, including the mountainous regions of northwest Saudi Arabia, central Ethiopia, southern Mexico, the central United States, and eastern Canada. Rainfall, the primary driver of soil erosion, shaped the erosion distribution (Fig 13a). However, exceptions include southwest China and parts of Russia, revealing that, despite significant increases in rainfall, soil erosion did not escalate to the same extent.

Regions witnessing significant rainfall increases in Europe exhibited enhanced vegetation coverage. This study provides evidence that the karst regions of China and Russia boast some of the highest rates of vegetation greening globally [53]. The NDVI index in China’s karst regions and central Russia experienced a significant rise during the study period (Fig 13b), likely attributed to ecological engineering policies in these regions.

Scenario analysis in Southwest China showed that roughly 72.65% of land use had a negative influence on soil erosion, demonstrating the moderating effect of changes in land use on erosion. Research has indicated a strong correlation between the decrease in soil erosion and the expansion of vegetation coverage [54], the Chinese government’s program of converting cropland back into forests has significantly enhanced the soil’s ability to retain water [55].

In addition to factors including soil moisture evaporation caused by rising temperatures, physiological drought occurring in plants, and limiting plant photosynthesis and growth rates, it is related to factors including urban area expansion [56], vegetation reduction, and large changes in land-use changes. This study’s analysis of the soil erosion trend reveals a decreasing trend in soil erosion in Europe from southwest to northeast, supported by existing research [57]. The decline in soil erosion amount and speed in Russia is related to climate trends, including the decrease in maximum soil freezing depth, maximum spring runoff flow, and flood runoff layer thickness. The long-term trend of river flow redistribution indicates that the surface runoff of cultivated slope land decreases sharply, and the surface runoff almost stops during spring snowmelt, consequently stopping meltwater erosion [58]. In comparison to Russia and China, Saudi Arabia in the Middle East and Ethiopia in Africa exhibit distinct eco-geographical characteristics. Saudi Arabia is characterized by a desert environment with limited water availability and low precipitation, with the majority of its terrain consisting of sandy soils [59,60]. In recent years, vegetation cover has diminished due to the rapid expansion of irrigated agricultural land and urban development. Additionally, the arid environmental conditions impose stress that may reduce soil moisture availability and increase plant mortality, thereby contributing to the formation of bare land areas. Notably, when soil exposure exceeds 30%, there is a marked escalation in soil erosion rates [61]. Agricultural activities are the backbone of Ethiopia’s economy, but soil erosion represents a significant environmental challenge to the sustainable agricultural development of Ethiopia [62]. National estimates indicate an annual soil loss of approximately 1.5 billion tonnes [63]. Empirical evidence suggests that forested areas in Ethiopia have been increasingly converted to food crop cultivation [64]. This trend, driven by rapid population growth, land scarcity, and heightened food production demands, has accelerated land-use changes throughout much of the Ethiopian Highlands [65]. Moreover, deforestation and agricultural expansion have reached unprecedented levels in recent centuries [62]. The consequent rapid reduction in forest cover has substantially increased surface runoff and soil degradation, thereby exacerbating the rate of soil erosion across the country.

A growing number of studies now show that extreme climate events, such as global greenhouse effects and droughts, are becoming more frequent. Climate change has also led to significant fluctuations in regional water resources and temperatures, putting considerable pressure on karst ecosystems [66,67]. The temperature increases caused by drought have had a profound impact on environmental changes in karst areas across the United States, Western Europe, Africa, and central South America [68]. Southeastern North America is one of the world’s three major regions rich in karst formations, with karst landscapes covering approximately 1,502,700 square kilometers. Our research indicates that the karst region in southeastern North America is strongly influenced by both climate change and human activities. Studies reveal that urban growth in the eastern United States is substantial, and this urbanization has triggered various environmental problems, including soil erosion, drought, and water shortages [69]. Rapid urban expansion has accelerated karst rocky desertification, leading to widespread exposure of bedrock and severe soil degradation. Moreover, drought has limited groundwater storage in bedrock and reduced plant survival, causing land use in the North American karst region to shift from forested areas to shrublands [70], which has further exacerbated soil erosion issues.

### Analysis of influencing factors of soil erosion

Soil erosion in karst landscapes is influenced by the complex interplay between anthropogenic activities and natural environmental factors. Changes in land use driven by human interventions account for the spatial variability observed in karst soil erosion patterns [71]. This research highlights the increasingly significant role of human activities in exacerbating soil erosion, particularly through extensive deforestation and human-initiated vegetation restoration efforts. Unsustainable human practices can precipitate land degradation and soil depletion. Over recent decades, deforestation in karst-dense regions of the Indochinese Peninsula has led to diminished vegetation cover [72]. In countries such as Myanmar, Thailand, and Cambodia, where agriculture constitutes a primary economic activity, local populations often clear shrubs and trees for crop production or engage in the burning of hillside vegetation, actions that disrupt soil structure and stability [73]. Conversely, anthropogenic ecological restoration initiatives offer promising strategies for mitigating soil erosion in karst areas. Enhanced vegetation cover resulting from these projects fosters conditions conducive to soil formation, thereby reducing soil loss [55]. At present, the ecological environment faces considerable challenges arising from global climate change. Climate change influences soil erosion through multiple mechanisms, such as alterations in plant biomass production and decomposition, variations in evapotranspiration rates, and changes in soil microbial activity [74]. Among these factors, the most direct and pronounced effect is the modification of rainfall erosivity. The intensity of rainfall governs both runoff generation and the extent of soil erosion. In karst landscapes, characterized by inherently thin soil layers, intense precipitation events more readily lead to the erosion and degradation of topsoil, thereby intensifying soil erosion processes [75]. Nonetheless, vegetation cover serves a critical function in intercepting rainfall, thereby playing a vital role in the preservation of karst soils.While the climate and vegetation cover can fluctuate dramatically in a short period, the terrain and soil qualities stay mostly constant [76]. In the correlation analysis of this study, the relationship between the NDVI and interannual soil erosion change exhibited spatial heterogeneity. Apart from the influence of extreme weather events, vegetation restoration may lead to decreased soil moisture [77], increased leaf area, deeper root systems, and higher aerodynamic roughness of vegetation, raising evapotranspiration rates and potentially forming dry soil layers, thereby contributing to regional vegetation degradation [78].

Previous research indicates that the response of vegetation activities at a regional scale relies on the spatial variability of climatic conditions and the background environment [79,80]. In karst regions, soil formation is slow, and the environment is unfavorable for plant growth, resulting in fragile ecosystems and a high frequency of soil erosion [81]. Vegetation in karst regions affects soil erosion through multiple pathways. Firstly, the vegetation canopy can significantly reduce the falling speed of raindrops, thereby minimizing direct damage to the topsoil and preventing soil disaggregation. Secondly, the network structure of vegetation roots enhances soil aggregate stability and thus improves erosion resistance. In addition, vegetation can reduce the frequency and intensity of surface gully erosion by intercepting rainfall, delaying surface runoff, and increasing rainfall infiltration [82]. Meanwhile, there is a significant interaction between karst vegetation and water. Vegetation alters surface infiltration conditions and the spatiotemporal distribution of water, while soil moisture conditions in turn affect vegetation growth [83]. For example, under high rainfall conditions during the rainy season, dense vegetation can buffer short-term runoff peaks through interception and enhanced infiltration, thereby alleviating erosion. However, under prolonged drought or seasonal dry periods, reduced vegetation coverage exposes bare land, leading to more severe erosion during heavy rainstorms [84]. The dissolution depressions and fracture systems of karst landforms also form complex and rapid infiltration pathways and local confluence zones, resulting in strong spatial heterogeneity of rainfall and surface water, which in turn amplifies local erosion [85]. Additionally, enhanced soil infiltration capabilities during vegetation restoration were observed due to improved physical and chemical soil properties, and the effects of root growth exerting pressure on and penetrating the soil were observed. This can lead to faster soil moisture accumulation during rainfall, reducing soil friction and increasing the risk of collapse and landslides [86]. Some laboratory studies suggest that vegetation restoration may increase landslide areas under heavy rainfall while decreasing collapse and mudflow volumes [9]. In summary, the erosion of karst soils is influenced primarily by climatic variables, notably precipitation, and is significantly modulated by vegetation composition and soil moisture conditions. These factors interact through mechanisms such as runoff redistribution, the stability of soil aggregates, and root reinforcement.

Several studies have highlighted that under annual mean conditions, the disparity and variability between surface and near-surface air temperatures in karst regions surpass those seen in places without karst [87]. Additionally, the stability of land-atmosphere energy exchange is significantly greater in karst areas compared to non-karst areas. In the correlation analysis conducted in this study, surface temperature exhibited a robust relationship with modifications to soil erosion in the designated regions. When contrasting the distribution of land use categories with the correlation map between soil erosion and surface temperature, a conspicuous negative correlation is evident for grassland and scrub meadow types around the region approximately 30 degrees north and south latitude.

Several potential explanations account for this phenomenon. First, vegetation litter biomass and nutrient replenishment rates in karst regions were positively correlated with maximum temperatures [88]. Elevated average annual temperatures expedite rock weathering, facilitate the turnover of soil microorganisms, enhance vegetation productivity [89], and notably foster the growth of plant roots. and foster plant root growth. Higher temperatures may also result in a drop in soil moisture content and an increase in evapotranspiration rate, thereby increasing the soil infiltration capacity and reducing soil erosion [90]. The dense root systems of grasslands effectively stabilize the soil and mitigate erosion. the rate of soil quality improvement increased with increasing regional temperature and precipitation [91]. Second, within areas sharing identical surface coverage, regions characterized by humid and sub-humid climates typically experience higher precipitation and intensity level [92,93]. Given that the growing season is longer and there is more vegetation cover, the increase in temperature inhibited soil erosion [94].Conversely, regions where soil erosion demonstrates a negative correlation with temperature primarily encompass tropical savannah climates, where temperature increases may mitigate the risk of soil erosion [95,96].

Soil erosion represents a significant environmental challenge. Numerous investigations have assessed soil erosion through various soil loss models, demonstrating that the intensity of soil erosion is largely influenced by natural processes [97,98]. Additionally, some research has highlighted that in karst regions, anthropogenic land-use alterations and the degradation of surface vegetation contribute to conditions that exacerbate subsurface soil loss [99]. Nevertheless, the majority of these studies have been confined to small-scale karst areas, lacking a comprehensive global perspective on karst soil erosion. In the present study, we integrated the revised Revised Universal Soil Loss Equation (RUSLE) with the Integrated Valuation of Ecosystem Services and Tradeoffs (InVEST) model. This approach enhances the depth of regional analyses and captures the dynamic characteristics of global karst soil erosion at a macro scale, systematically quantifying the relationships among soil erosion, climate change, and human activities across an extensive spatial domain.The results of this study’s model are consistent with the soil erosion results from the European Soil Data Centre (ESDAC) [100]. However, the results from 2001 are generally higher than the ESDAC 2001 soil erosion results, primarily determined by the characteristics of the raw data input into the model. Traditional runoff plot observation data are reliable but require significant investment and time. They are influenced by area, soil thickness, topography, slope, aspect, and vegetation cover, making it challenging to reflect soil erosion conditions over large areas. Therefore, they can serve as a means to verify model calculation methods. The results of this study are consistent with existing studies in small watersheds in karst regions of Brazil [101] and China [12], demonstrating the reliability of the research results.

### Uncertainty and implication

Many factors influence soil erosion. Although the correlation and scenario analyses adopted in this study can generally quantify the impacts of climatic and anthropogenic factors on soil erosion, they do not fully elaborate on the interactive effects among all climatic, anthropogenic, and vegetation factors. This constitutes a major source of uncertainty in this study, and data precision often influences model calculation methods. The resolution precision of the input model affects the accuracy of the data results, and the calculation methods selected for each input factor also affect the calculation results, such as the choice of different rainfall formulas. Annual rainfall amounts may overlook some details in the calculation of soil erosion [102], and the calculation of the K factor takes into account the coarse particles of karst soils. Additionally, due to the lack of global rainfall intensity data, this study only used rainfall amounts to calculate rainfall erosion force. In karst regions, soil and water loss exhibit a distinct “dual structure” of surface and subsurface loss [103]. As desertification intensifies, surface erosion becomes less apparent, and soil erosion primarily shifts to subsurface loss [104]. However, this aspect of soil erosion was not addressed in this study. Moreover, the utilization of diverse remote sensing datasets may impact the research outcomes; hence, incorporating additional remote sensing data, such as the widely employed GIMMS-NDVI dataset for monitoring vegetation dynamics, is recommended to enhance and validate the findings of this study [105]. Additionally, the integration of extended meteorological datasets, for instance, high-resolution long-term (HRLT) daily data, should be thoroughly considered. [106] Finally, various other factors, including soil texture and organic matter content, also play significant roles in karst soil erosion. Consequently, further investigation into the mechanisms by which these factors influence karst soil erosion is necessary to comprehensively elucidate the evolutionary patterns of soil erosion in karst environments.

## Conclusions

This study applied the InVEST model, combined with the Theil-Sen and Mann-Kendall test, to analyze the spatiotemporal characteristics of soil erosion across global karst regions from 2000 to 2020, while quantifying the absolute contributions of major countries to soil erosion reduction. Pearson correlation analysis and scenario-based analysis were employed to evaluate the differential responses of soil erosion to various influencing factors and to assess the contribution of each factor. The findings demonstrate a global decline in soil erosion, with 76.59% of the studied area exhibiting a downward trend and 5.81% showing a statistically significant reduction. This decrease was predominantly observed in Asia and Europe, whereas North America experienced an increasing trend. Notably, Russia, China, and Europe significantly contributed to the mitigation of global soil loss, accounting for 29.98%, 28.01%, and 18.68% of the total reduction, respectively. Conversely, the United States had a detrimental effect on the reduction of global soil erosion, with an absolute contribution rate of −9.21%. Correlation and scenario analyses indicate that soil erosion in global karst regions is primarily influenced by precipitation and land use changes, while its association with vegetation coverage and land surface temperature is comparatively weak.These findings provide theoretical guidance and technical support for understanding the current status of global karst soil erosion and for implementing scientific and sustainable soil erosion control and reduction projects.
